# Automated size‐specific dose estimates framework in thoracic CT using convolutional neural network based on U‐Net model

**DOI:** 10.1002/acm2.14283

**Published:** 2024-01-31

**Authors:** Sakultala Ruenjit, Punnarai Siricharoen, Kitiwat Khamwan

**Affiliations:** ^1^ Medical Physics Program, Department of Radiology Faculty of Medicine Chulalongkorn University Bangkok Thailand; ^2^ Division of Diagnostic Radiology Department of Radiology King Chulalongkorn Memorial Hospital The Thai Red Cross Society Bangkok Thailand; ^3^ Chulalongkorn University Biomedical Imaging Group Depertment of Radiology, Faculty of Medicine Chulalongkorn University Bangkok Thailand; ^4^ The Perceptual Intelligent Computing Lab Department of Computer Engineering Faculty of Engineering Chulalongkorn University Bangkok Thailand; ^5^ Division of Nuclear Medicine Department of Radiology Faculty of Medicine Chulalongkorn University Bangkok Thailand

**Keywords:** convolutional neural network, size‐specific dose estimate, thoracic CT, U‐Net model

## Abstract

**Purpose:**

This study aimed to develop an automated method that uses a convolutional neural network (CNN) for calculating size‐specific dose estimates (SSDEs) based on the corrected effective diameter (D_eff_^corr^) in thoracic computed tomography (CT).

**Methods:**

Transaxial images obtained from 108 adult patients who underwent non‐contrast thoracic CT scans were analyzed. To calculate the D_eff_^corr^ according to Mihailidis et al., the average relative electron densities for lung, bone, and other tissues were used to correct the lateral and anterior–posterior dimensions. The CNN architecture based on the U‐Net algorithm was used for automated segmentation of three classes of tissues and the background region to calculate dimensions and D_eff_^corr^ values. Then, 108 thoracic CT images and generated segmentation masks were used for network training. The water‐equivalent diameter (D_w_) was determined according to the American Association of Physicists in Medicine Task Group 220. Linear regression and Bland–Altman analysis were performed to determine the correlations between SSDE_Deff_^corr(automated)^, SSDE_Deff_^corr(manual)^, and SSDE_Dw_.

**Results:**

High agreement was obtained between the manual and automated methods for calculating the D_eff_^corr^ SSDE. The mean values for the SSDE_Deff_^corr(manual)^, SSDE_Dw_, and SSDE_Deff_^corr(automated)^ were 14.3 ± 2.1 mGy, 14.6 ± 2.2 mGy, and 14.5 ± 2.4 mGy, respectively. The U‐Net model was successfully trained and used to accurately predict SSDEs, with results comparable to manual‐labeling results.

**Conclusion:**

The proposed automated framework using a CNN offers a reliable and efficient solution for determining the D_eff_^corr^ SSDE in thoracic CT.

## 1 INTRODUCTION

Computed tomography (CT) scans deliver a higher dose of ionizing radiation than that of other imaging modalities, which presents a potential health risk to patients.^1^, ^2^, ^3^ Implementation of protocol optimization is an essential approach to minimizing potential risks associated with exposure to ionizing radiation while still achieving the required diagnostic information.^4^, ^5^ Accurate dose estimation enables physicians to weigh the examination benefits against the potential harm to the patient and make informed decisions about the use of CT examinations.^6^, ^7^, ^8^ In 2011, the American Association of Physicists in Medicine (AAPM) Task Group 204 (TG204) published a patient‐specific dose descriptor based on the size of the patient called the size‐specific dose estimate (SSDE).^9^ The SSDE calculation involves multiplying the volumetric CT dose index (CTDI_vol_, expressed in milligray) by the patient size‐dependent conversion factor (f_size_). However, the SSDE calculation using geometric parameters according to the TG204 report does not account for the patient's composition or tissue properties that affect radiation attenuation.^10^ Subsequently, the methodology described in the AAPM TG220^11^, ^12^ addresses the patient size estimation limitation in CT scans by using the water‐equivalent diameter (D_w_) concept to improve the SSDE accuracy. Although several patient dose‐monitoring programs can provide the SSDE based on either the effective diameter or D_w_, the SSDE is still not readily accessible in the patient dose report generated by a CT scanner.^13^, ^14^

Recently, Mihailidis et al.^15^ proposed a method for calculating the patient diameter that uses x‐ray attenuation properties to correct the effective diameter for estimating the D_w_ used in the SSDE calculation in chest CT. The results of this method, which only uses the lateral thickness, were comparable to those from the D_w_‐based calculation. Despite those promising findings, that study had some limitations. First, an average relative electron density was scaled only for the lung and other tissues that may affect the measurement accuracy. Second, the sample size in the study was small, thus limiting the generalizability of the results. Finally, the tissues in each segment had to be manually measured for calculating the corrected effective diameter (D_eff_^corr^), which is time‐consuming and prone to high inconsistency. These limitations indicate that further research is needed to modify and/or validate the method before it can be widely adopted in clinical practice. Additionally, an automated measurement approach would have advantages over the manual method.

Currently, the use of deep learning algorithms for image segmentation has become a popular approach in medical imaging due to its ability to learn complex features from the provided data and generate accurate results.^16^, ^17^, ^18^, ^19^ Semantic segmentation, which involves pixel‐wise image segmentation, is a well‐investigated problem in computer vision and uses deep learning algorithms. The goal of semantic image segmentation is to classify each image pixel into one of a defined set of classes, resulting in the classification of various entities in the image. Numerous models have been developed for the task of semantic image segmentation, and an appropriate model architecture should be selected according to the particular applications.^20^, ^21^, ^22^ To the best of our knowledge, no previous studies have described an automated method for calculating the SSDE based on the approach proposed by Mihailidis et al. This study aimed to develop and test a proposed automated framework using a U‐Net convolutional neural network (CNN)^21^ to determine the SSDE in chest CT with the goal of reducing measurement variability and to assess the correlation between the SSDE determined using the D_eff_^corr^ and using the D_w_.

## 2 MATERIAL AND METHODS

### 2.1 Study population

This study was conducted in compliance with protocols approved by the institutional review board and included a retrospective analysis of data from 108 adult patients (52 males, 56 females) who underwent thoracic CT scans. The patients had a mean age of 64.3 ± 15.4 years (range: 22−93 years) and a mean weight of 58.7 ± 11.8 kg (range: 33.6−89.0 kg). For each patient, a slice with full field‐of‐view (FOV) reconstructions and no tissue truncation was selected, and sets of central axial images were used for body‐diameter measurements.

### 2.2 Imaging protocols

All patients underwent non‐contrast thoracic CT scans on a 64‐slice GE Discovery CT750 HD scanner with a standardized protocol. The following imaging settings were used: tube voltage: 120 kVp; automated tube‐current modulation (TCM); slice thickness: 1.25 mm; pitch factor: 1.375; rotation time: 0.4 s; noise index: 14; and scan‐range encompassing the lung apices to the adrenal glands. The images were reconstructed with a filtered back projection (FBP) algorithm, a 512 × 512 matrix, 5‐mm slice thickness and interval, and a 36‐cm display FOV. The CT reconstructed image series was automatically transferred to a Picture Archiving and Communication System in Digital Imaging and Communications in Medicine (DICOM) format.

### 2.3 Manual approach

**FIGURE 1:**
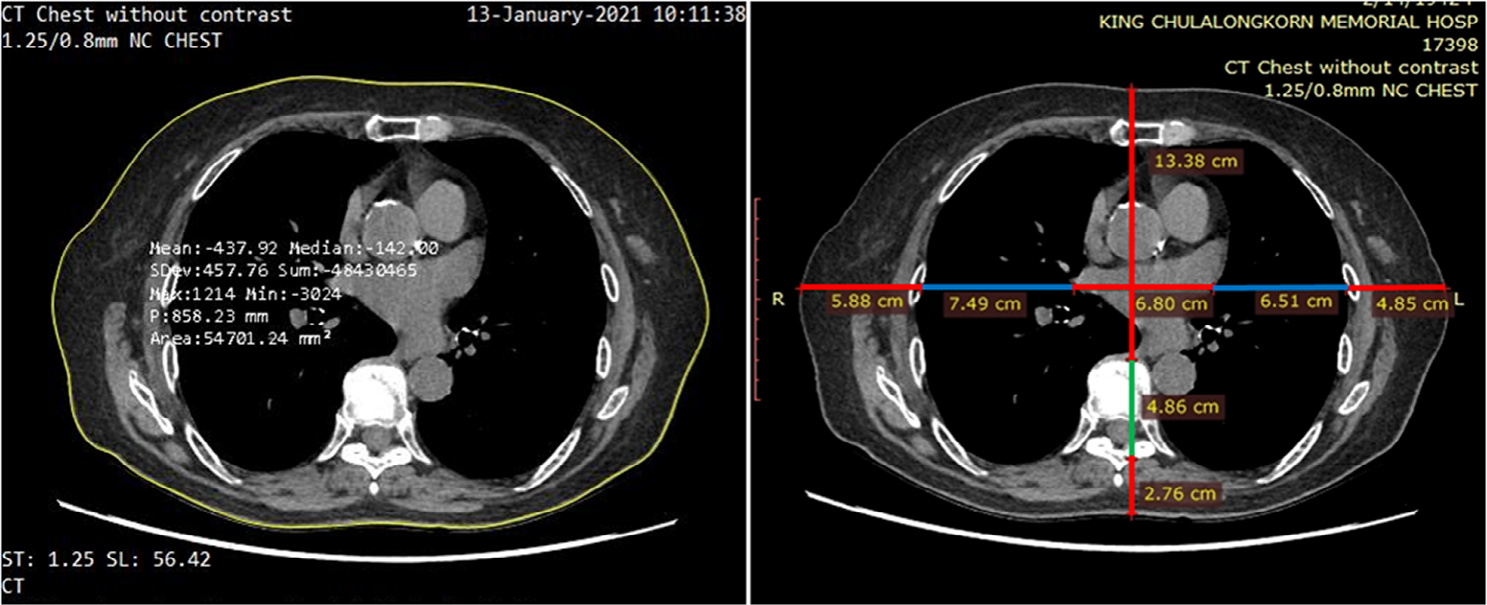
(Left) Manual contouring on the transaxial image for the water‐equivalent size‐specific dose estimate calculation (DICOM viewer software). (Right) Manual measurement of the anterior–posterior and lateral dimensions along with the individual distance of lung tissue (blue line), other tissues (red line), and bone (green line) for relative electron density correction. DICOM, Digital Imaging and Communications in Medicine.

To derive the SSDE, two methods were used to measure each patient image. First, the D_w_ was determined according to the AAPM TG220.^11^, ^12^ The region of interest (ROI) based on the patient body contour was manually drawn on the axial image to determine the mean CT number and entire area inside the ROI, and the D_w_ value was then calculated using the following equation^11^, ^12^:

(1) $$\mathrm{Dw}\left(\mathrm{cm}\right)=2\sqrt{\left[\frac{1}{1000}\bar{CT{\left(x,y\right)}_{ROI}}+1\right]\frac{A_{ROI}}{\pi }}$$

where, $\bar{CT{\left(x,y\right)}_{ROI}}$ is the mean CT number in the ROI, and $A_{ROI}=\sum A_{pixel}$ is the total area in the ROI. Second, the D_eff_^corr^, as defined in accordance with Mihailidis et al., was calculated. According to the method published by Mihailidis et al., the D_eff_^corr^ was obtained only in the lateral thickness, and the average relative electron density was scaled for two regions in lung (ρ_e_^lung^ = 0.3) and other tissues (ρ_e_^tissue^ = 1.0).^15^, ^23^ To improve the calculation accuracy in this study, the thicknesses of water‐equivalent tissue in the anterior–posterior (AP) and lateral (LAT) dimensions were determined, and the average relative electron density for bones (ρ_e_^bone^ = 1.2),^23^ in addition to that of lungs and other tissues, was taken into account. Each relative electron tissue density was multiplied by the length of each segment, and then all were summed to give the patient dimension, as calculated according to Equation (2). The D_eff_^corr^ was calculated as the corrected AP ($AP_{eff}^{corr}$) and corrected LAT ($LAT_{eff}^{corr})$ dimensions according to Equation (3)^15^:

(2) $$Dimension\left(cm\right)=\sum_{j=1}^{N_{s}}\rho_{e}^{s}\left(j\right)\times l_{s}\left(j\right)$$

(3) $$D_{eff}^{corr}\left(cm\right)=\sqrt{AP_{eff}^{corr}x\;LAT_{eff}^{corr}}$$

where $\rho_{e}^{s}\left(j\right)$ is the density of the tissue relative to water of the j‐segment, $l_{s}\left(j\right)$ is the length of the j‐segment, with the subscript “s” referring to the tissue segment and superscript N_s_ indicating the number of line segments along the dimension of interest. The illustration of contouring on the transaxial image for the water‐equivalent SSDE calculation and the measurements of the AP and LAT dimensions along with the individual distance for the D_eff_^corr^ calculation are shown in Figure 1.

### 2.4 Automated‐SSDE calculation framework

**FIGURE 2:**
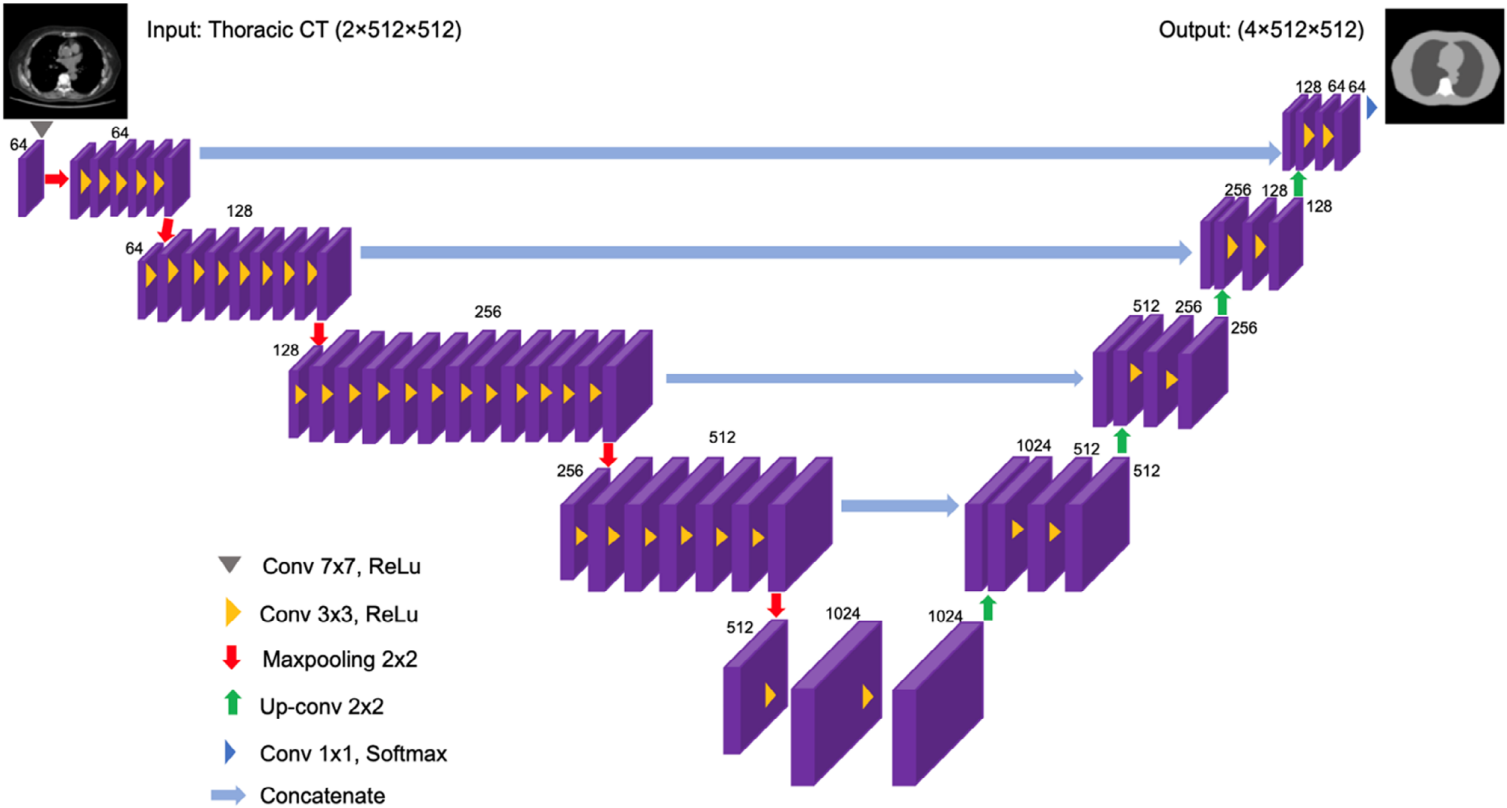
U−Net architecture with ResNet34 blocks in the down‐sampling path for automated segmentation of the three classes of tissues and the background region used in the corrected effective diameter calculation.

To develop an automated framework for the SSDE calculation, the initial step involved the utilization of a Python‐based CNN algorithm. This algorithm was developed using the Keras library and was implemented within a Collaboratory notebook to perform automatic segmentation of tissues in the axial thoracic CT images. The CNN architecture used the U‐Net with Resnet34 blocks in the down‐sampling path to achieve the desired segmentation.^21^ Pre‐trained models from the ImageNet dataset were used as the starting point to enhance the training process and improve the model's performance.^24^, ^25^ The CNN architecture that was designed for automated segmentation of the three classes of tissues (lung, bone, and other tissues) and the background region used in calculating dimensions and the D_eff_^corr^ is shown in Figure 2.

The input layer consists of images with a matrix size of 512 × 512. The original CT slices were resized through a down‐sampling path, which consisted of five blocks and incorporated 34 convolutional layers from the original ResNet34. Each convolution block was followed by a rectified linear unit (ReLU) activation function and a 2 × 2 max pooling operation with a stride of 2, which reduced the image's spatial dimension. The network included a 2 × 2 up‐sampling path, as depicted on the right side of the architecture. The skip connections between the blocks were connected with the down‐sampling and up‐sampling paths, and the up‐sampling path was allowed to use the high‐resolution feature maps from the early stages of the network without losing information through pooling, thus restoring the image dimensions. A concatenation operation was used to combine the corresponding feature maps to implement the connections.^21^ The final layer was a 1 × 1‐convolutional layer, followed by the Softmax activation function, which outputs the four‐class mask prediction of the thoracic image, including the background, lung, soft tissue, and bone.

**FIGURE 3:**
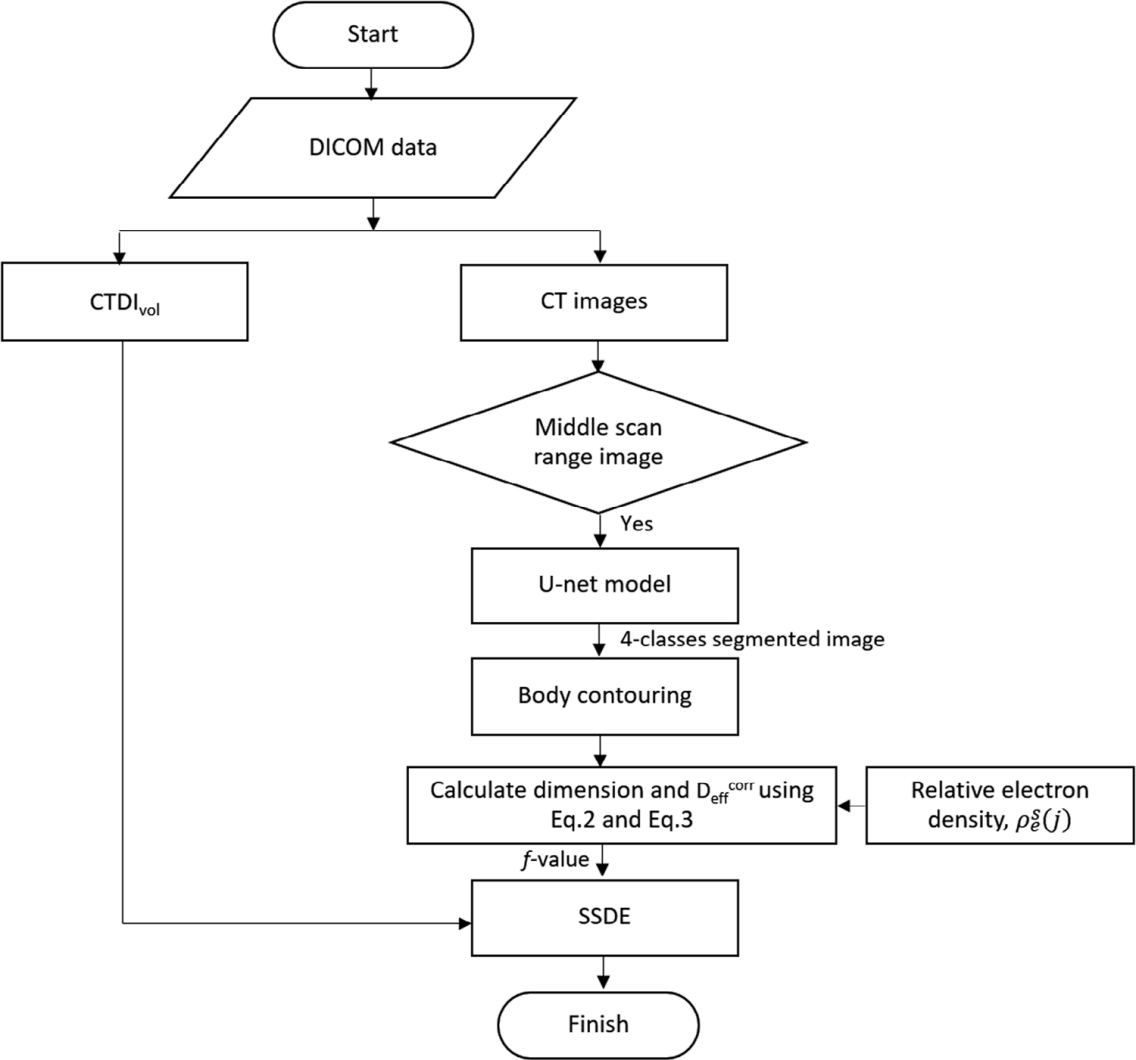
Flowchart of the automated SSDE based on the corrected effective diameter (D_eff_^corr^) framework. SSDE, size‐specific dose estimate.

An overview of our automated SSDE calculation framework is presented in Figure 3. DICOM data extracted from the CT images, including the pixel size, CTDI_vol_, and other attributes, were entered as input data. To train the CNN model for the D_eff_^corr^ SSDE in thoracic CT images, 108 input images and their respective segmentation masks were used. The network was trained for 200 epochs, with 20% of the data set aside for testing. The Jaccard Similarity Index, also known as the Intersection over Union (IoU),^26^ was used to assess the similarity between the manual label and predicted masks, providing a measure of the model's prediction accuracy. After using the segmented lungs, bone, and other tissue regions to extract the body contours, the major and minor body axes were determined from the contours. The AP and LAT dimensions, calculated using Equation (2), were derived from the major and minor axes and the average relative electron density of the different tissues that had been segmented by the U‐Net model. D_eff_^corr^ was computed according to Equation (3), the *f*‐value was computed according to Equation (5) and then SSDE was computed according to Equation (4).

### 2.5 Calculation of SSDE based on the D_eff_^corr^ and D_w_

The SSDEs (SSDE_Deff_^corr (manual)^, SSDE_Dw_^(manual)^, and SSDE_Deff_^corr (automated)^), which were derived from the manual D_eff_^corr^, manual D_w_, and automated D_eff_^corr^, were calculated according to the following equation^9^, ^10^, ^11^, ^12^:

(4) $$\mathrm{SSDE}\left(\mathrm{mGy}\right)=f\times {\mathrm{CTDI}}_{\mathrm{vol}}$$

where *f* is the size‐dependent conversion factor to correct patient size based on the D_eff_^corr^ and D_w_. The CT dose descriptor in terms of CTDI_vol_ reported by the scanner is the average CTDI_vol_ across all slices of the scan range. The CTDI_vol_ is calculated on the basis of measurements made in a standard polymethyl methacrylate (PMMA) cylindrical phantom of a specified size with either a 16‐cm head or a 32‐cm body phantom to quantify the x‐ray tube output emitted by the scanner.^6^, ^7^, ^8^ Since a tube voltage of 120 kVp was used, the *f* value was calculated according to Equations (5) and (6) as follows^11^, ^12^, ^15^:

(5) $$f=3.704369\times e^{-0.03671937\times D_{\mathrm{eff}}^{\mathrm{corr}}}$$

(6) $$f=3.704369\times e^{-0.03671937\times D_{w}}$$

where D_eff_^corr^ and D_w_ are patient size specific expressed in centimeters.

### 2.6 Validation

The results of the automated SSDE were compared with those obtained through manual scaling of tissues. Regression analysis was performed to examine the correlation between the SSDE calculated by the automated method, manual D_eff_^corr^ method, and D_w_ method. Pearson's correlation test was performed to determine the agreement between the D_eff_^corr^ SSDE and D_w_ SSDE calculated values. The percentage difference between the automated and manual D_eff_^corr^ SSDEs was calculated, and the accuracy of the comparison was evaluated by performing the Bland–Altman analysis.

## 3 RESULTS

The measurement of all manual D_eff_^corr^, automated D_eff_^corr^, and D_w_ values were <32 cm in diameter, and all values of the size‐dependent conversion factors based on the manual D_eff_^corr^ (*f*_Deff_^corr(manual)^), automated D_eff_^corr^ (*f*_Deff_^corr(automated)^), and manual D_w_ (*f*_Dw_) were >1. The mean values of CTDI_vol_, SSDE_Deff_^corr(manual)^, SSDE_Deff_^corr(automated)^, and SSDE_Dw_ were 8.5 ± 1.7 (range: 4.2−10.5) mGy, 14.3 ± 2.1 mGy (range: 8.5−17.9) mGy, 14.5 ± 2.4 mGy (range: 8.0−17.9) mGy, and 14.6 ± 2.2 (range: 8.6−18.2) mGy, respectively. The results presented in Table 1 show overall agreement between the D_eff_^corr^ and D_w_, with both SSDE_Deff_^corr^ values (obtained using the manual and automated methods) being slightly lower than the SSDE_Dw_. The SSDE values obtained from the three methods for male and female subjects are presented in Table 2.

**TABLE 1:** The SSDE (mGy) results obtained from the three calculation methods.

Approach	Body size (cm)	CTDI_vol_ (mGy)	*f*	SSDE (mGy)
Manual SSDE−D_eff_^corr^	20.9 ± 2.4 (15.9−25.9)	8.5 ± 1.7 (4.2−10.5)	1.7 ± 0.2 (1.4−2.1)	14.3 ± 2.1 (8.5−17.9)
Automated SSDE−D_eff_^corr^	20.7 ± 1.9 (16.3−26.7)	8.5 ± 1.7 (4.2−10.5)	1.7 ± 0.1 (1.4−2.1)	14.5 ± 2.4 (8.0−17.9)
Manual SSDE−D_w_	20.4 ± 2.1 (15.9−25.4)	8.5 ± 1.7 (4.2−10.5)	1.8 ± 0.1 (1.5−2.1)	14.6 ± 2.2 (8.6−18.2)

Abbreviations: Automated SSDE‐D_eff_^corr^, SSDE based on automatic calculation of the corrected effective diameter; *f*, size‐dependent conversion factor; Manual SSDE‐D_eff_^corr^, SSDE based on the manually‐measured corrected effective diameter; Manual SSDE‐D_w_, SSDE based on the manually‐measured water‐equivalent diameter; SSDE, size‐specific dose estimate.

**TABLE 2:** The SSDE (mGy) results obtained from the three calculation methods classified by sex.

Sex	Approach	Body size (cm)	CTDI_vol_ (mGy)	SSDE (mGy)
Male	Manual SSDE−D_eff_^corr^	21.6 ± 2.1 (17.1−25.9)	9.3 ± 1.2 (6.0−10.5)	15.4 ± 1.3 (11.5−17.9)
	Automated method	21.1 ± 1.9 (17.5−26.7)	9.3 ± 1.2 (6.0−10.5)	15.8 ± 1.5 (11.3−17.9)
	Manual SSDE−D_w_	21.0 ± 1.9 (17.1−25.4)	9.3 ± 1.2 (6.0−10.5)	15.8 ± 1.4 (11.9−18.2)
Female	Manual SSDE−D_eff_^corr^	20.4 ± 2.5 (15.9−25.6)	7.7 ± 1.8 (4.2−10.5)	13.3 ± 2.2 (8.5−17.4)
	Automated method	20.4 ± 2.0 (16.3−24.7)	7.7 ± 1.8 (4.2−10.5)	13.3 ± 2.4 (8.0−17.2)
	Manual SSDE−D_w_	19.9 ± 2.1 (15.9−24.9)	7.7 ± 1.8 (4.2−10.5)	13.6 ± 2.4 (8.6−17.2)

Abbreviations: Automated method, SSDE based on automatic calculation of the corrected effective diameter; Manual SSDE‐D_eff_^corr^, SSDE based on the manually‐measured corrected effective diameter; Manual SSDE‐D_w_, SSDE based on the manually‐measured water‐equivalent diameter; SSDE, size‐specific dose estimate.

**FIGURE 4:**
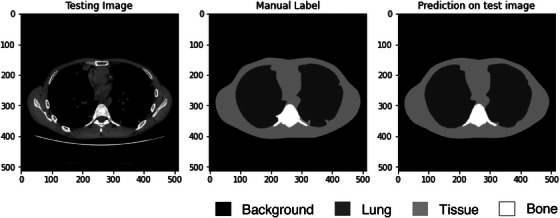
Results for automated segmentation using the U−Net model of the three classes of tissues and the background region shown in transaxial thoracic computed tomography images.

**FIGURE 5:**
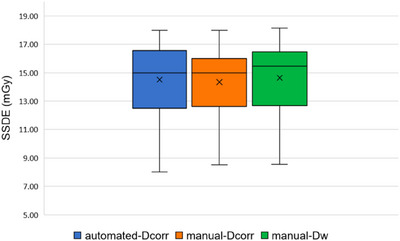
Box plots of the SSDE based on the three calculation methods. SSDE, size‐specific dose estimate

**FIGURE 6:**
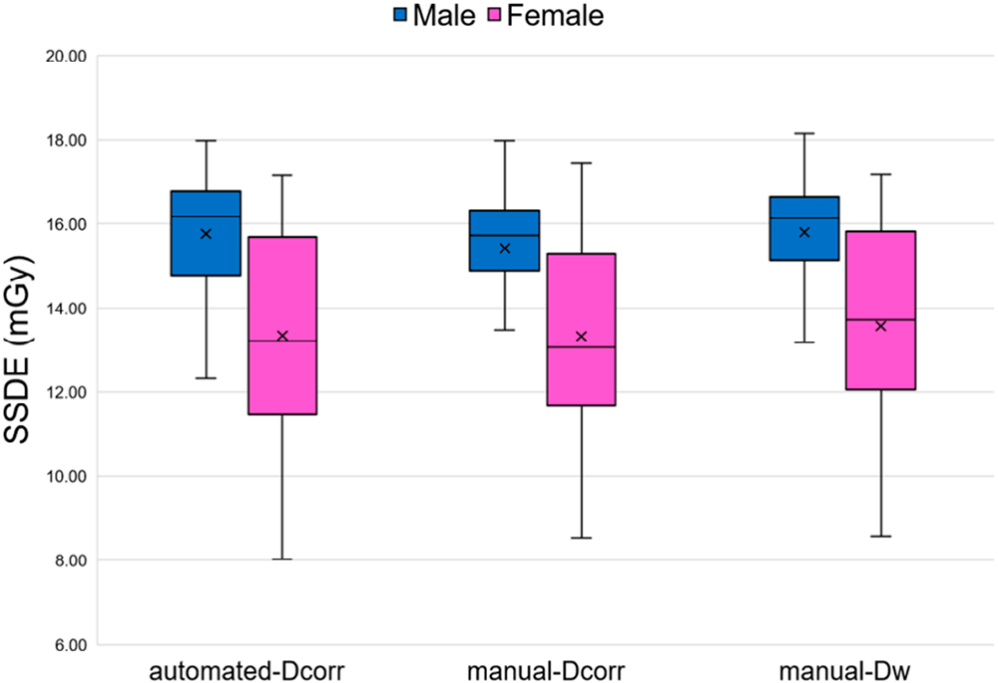
Box plots of the SSDE based on the three methods for males and females. SSDE, size‐specific dose estimate.

**FIGURE 7:**
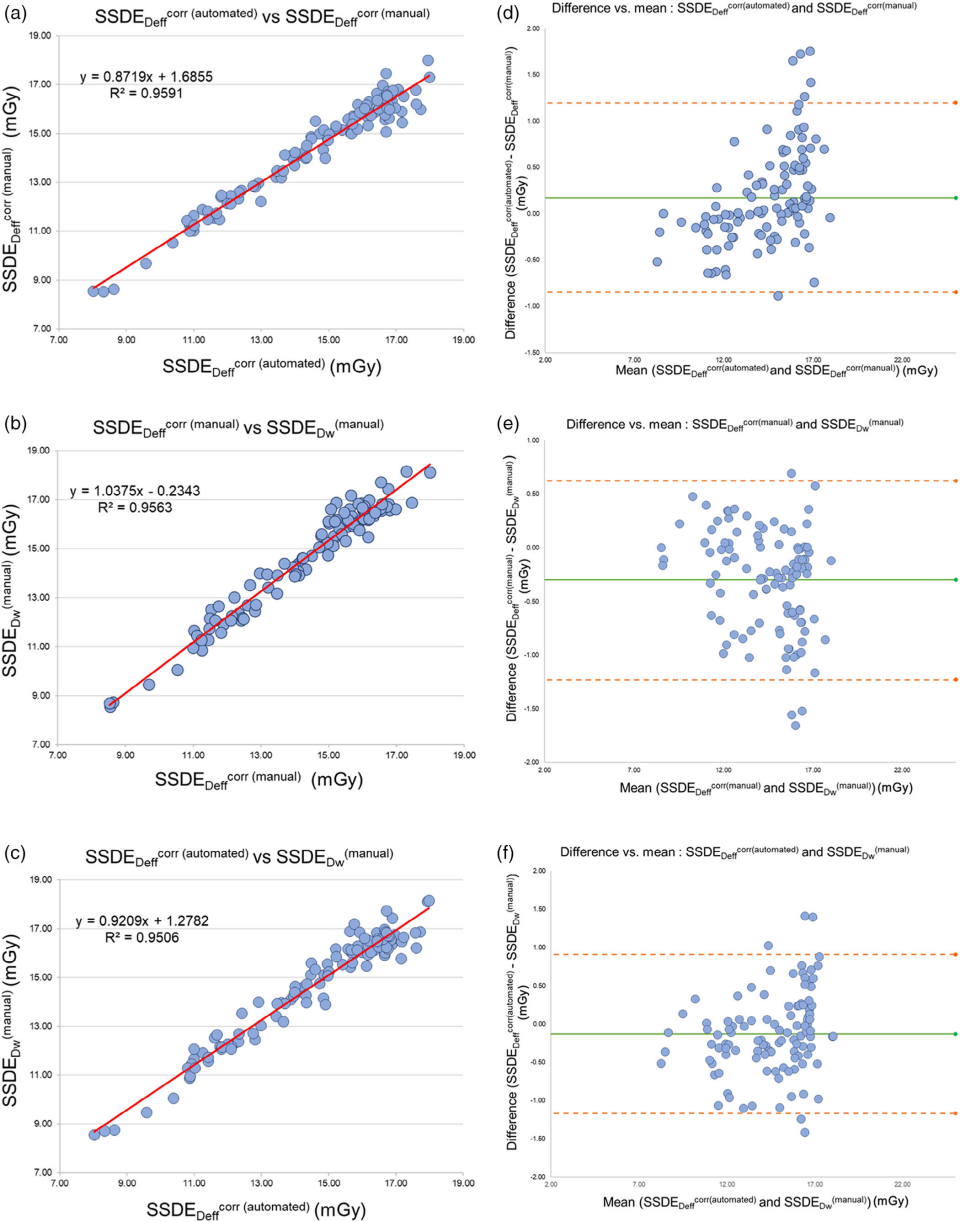
Scatter plots and Bland−Altman analysis showing the correlations between the SSDE calculated by the automated method, manually‐corrected effective diameter (D_eff_^corr^), and water‐equivalent diameter (D_w_). SSDE, size‐specific dose estimate.

The average IoU scores for the segmentation model across various tissue classes in thoracic CT images, including lung, bone, other tissues, and background, were satisfactory, with values of 0.96, 0.86, 0.95, and 0.99, respectively, averaging 0.94. Figure 4 illustrates the results of the CNN‐generated segmentation model, which predicted the testing thoracic CT image correctly when juxtaposed with the manually‐labeled ground truth comprising three classes of tissues and the background region. Figure 5 shows the box plots of the SSDEs based on the automated method, manual D_eff_^corr^, and D_w_ obtained in this study, and Figure 6 shows the SSDE results according to classification by sex. The validation study results show the correlations determined by linear regression (*R^2^*), Pearson's correlation coefficient (*r*), percentage difference, and Bland–Altman analysis of the SSDEs obtained from the three calculation methods (Table 3 and Figures 7a–f).

**TABLE 3:** The results of validation of the SSDE (mGy) obtained from the three calculation methods.

Approach	*R* ^2^	Pearson's correlation coefficient (*r*)	Average %difference	Largest %difference	Bland–Altman analysis
Automated−SSDE versus Manual SSDE−D_eff_^corr^	0.96	0.98	+1.0%	+11.2%	Good agreement
Manual SSDE−D_eff_^corr^ versus Manual SSDE−D_w_	0.96	0.98	−1.9%	−9.8%	Good agreement
Automated−SSDE versus Manual SSDE−D_w_	0.95	0.97	−1.0%	+8.9%	Good agreement

*Note*: %Diff = [(SSDE−D_eff_^corr(automated)^ − SSDE−D_eff_^corr(manual)^) / SSDE−D_eff_^corr(manual)^] × 100.

Abbreviations: Automated−SSDE, SSDE based on automatic calculation of the corrected effective diameter; Manual SSDE−D_eff_^corr^, SSDE based on the manually‐measured corrected effective diameter; Manual SSDE−D_w_, SSDE based on the manually‐measured water‐equivalent diameter; SSDE, size‐specific dose estimate.

## 4 DISCUSSION

Accurate estimation of radiation dose in patients undergoing CT scans requires precise measurement of patient size because the dose received depends on the patient factors, pitch factor, scan length, and CT scanner output that is influenced by the parameter settings.^4^, ^5^, ^6^, ^8^ Use of SSDE based on the SSDE_Deff_ is a straightforward approach to estimating radiation doses in CT scans. However, in regions with significant x‐ray attenuation inhomogeneities, the SSDE_Deff_ may result in an underestimation of patient size and, subsequently, an overestimation of SSDE.

In this study, we tested a proposed framework of automated SSDE_Deff_^corr^ that uses deep learning and takes into account the patient's attenuation properties for estimating the radiation dose received by patients during thoracic CT scans. Additionally, we considered the average relative electron density in both the AP and LAT dimensions to further improve the accuracy of the D_eff_^corr^, as described by Mihailidis et al.^15^ We found that both automated and manual SSDE_Deff_^corr^ methods had strong correlations with the SSDE_Dw_, as demonstrated by the results in Figures 7b,c, since we accounted for correction in both the AP and LAT dimensions in a larger patient dataset than that used by Mihailidis et al. The differences in the patient‐equivalent diameter between the automated SSDE_Deff_^corr^ method and SSDE_Dw_ (+13.2%) and between the manual SSDE_Deff_^corr^ and SSDE_Dw_ (+13.6%) in the present study were similar to the findings reported by Mihailidis et al. in which the discrepancy between the D_eff_^corr^ and D_w_ was +13.3%. In a study by Juszczyk et al.,^27^ who used an automated D_w_ to calculate the SSDE and compared the results to those determined by the GE DoseWatch, their mean SSDE was higher than the one in our study. This finding is probably because of the wider range of patient body sizes in Juszczyk et al.’s study, including a maximum chest diameter >32 cm, than those in our study. Juszczyk et al. reported a mean SSDE of 18.8 ± 14.3 (range: 3.4−64.8), whereas in our study, the mean SSDE values calculated by the three methods were smaller.

Considering the sex‐dependent results shown in Table 2, all SSDEs calculated by the three methods were higher for males than for females. This finding is probably because males generally have larger body sizes, particularly in the AP dimension, resulting in higher SSDE values (Figure 6). An automatic TCM system adjusts the tube current in the x–y plane (angular modulation), along the scanning direction (z‐axis modulation), or both (combined modulation) on the basis of the size and attenuation map of the body region being scanned to maintain consistent image quality while reducing radiation dose.^4^, ^5^, ^6^, ^8^ However, different CT scanners use different algorithms in their TCM system depending on the acquired projection radiograph. In case of longitudinal mA modulation, the tube current is mainly adjusted on the basis of the change in the AP thickness of the patient along the *z*‐axis. Since the AP diameter of the thoracic region of females differs significantly from those of males, females might benefit from scanning parameters using a lesser dose for this situation.

In this study, an automated method was used for accurate and consistent estimation of SSDE using a CNN and image processing. The U‐Net model with ResNet34 was used for image segmentation and achieved generally satisfactory results, with an accuracy of >90% for the four classes of segmented images. The IoU score for bone was slightly lower than for other classes due to its smaller region and more concave shape, which may have affected the D_eff_^corr^ determination. The accuracy of the model is crucial for SSDE calculations because any overestimation of D_eff_^corr^ may cause underestimation of the SSDE and vice versa. To enhance the model's accuracy, additional patient data is required to accommodate inter‐patient variability. Our validation of the automated SSDE_Deff_^corr^ method showed strong correlation with the manual method (Figure 7a). To address the issue of limited patient data, this study used transfer learning with a pre‐trained model on the ImageNet dataset. This approach allowed the model to effectively handle small datasets and still produce accurate results.^24^, ^25^ The deep U‐Net structure with a ResNet34 encoder was implemented to perform automated segmentation of CT images, which provided a useful starting point for the study.

This study had some limitations, including that the CT patient data were only retrieved from one institution and may need to be confirmed in a larger multi‐center dataset in future studies. Second, our method was not tested in pediatric patients, especially neonatal to ∼10‐year‐old patients. Third, because this study mainly used non‐contrast thoracic CT images for the SSDE calculation, the measurement accuracy for each segment may have affected the scans for those that used iodinated contrast, especially for the arterial‐phase contrast enhancement images in which the structures such as the vascular and parenchymal tissues will be markedly enhanced by the contrast agents, which may cause the CNN model to segment them as a bone class. Last, because our automated method did not include the ribs or sternum as bone in the training data, we may need to consider incorporating these bones into our method to improve automated segmentation in future research.

## 5 CONCLUSIONS

In this study, we tested a proposed novel method for calculating the SSDE_Deff_^corr^ in thoracic CT that uses a CNN based on a U‐Net model. The proposed framework achieved results comparable to those of the manual method while significantly reducing the time required for calculation and improving measurement consistency. These advantages potentially can enhance the clinical workflow and provide a valuable tool for patient dose estimation. The study results demonstrate that the SSDE_Deff_^corr^ can be used as a simple and efficient alternative for accurate SSDE calculation in thoracic CT, similar to the SSDE_Dw_, for estimating patient doses.

## AUTHOR CONTRIBUTIONS

Sakultala Ruenjit contributed to data collection, data analysis, software and validation, and manuscript drafting. Punnarai Siricharoen contributed to conception, software and validation, and manuscript drafting. Kitiwat Khamwan contributed to study design and conception, validation, and manuscript drafting.

## CONFLICTS OF INTEREST STATEMENT

The authors declare no conflicts of interest.
